# Role of Probiotics in the Management of Helicobacter pylori

**DOI:** 10.7759/cureus.26463

**Published:** 2022-06-30

**Authors:** Andrea Mestre, Rajalakshmi Sathiya Narayanan, Deliana Rivas, Jobby John, Mohammed Ali Abdulqader, Tushar Khanna, Raja Chandra Chakinala, Sachin Gupta

**Affiliations:** 1 Internal Medicine, Universidad del Rosario, Bogota, COL; 2 Internal Medicine, Stanley Medical College, Chennai, IND; 3 Internal Medicine, Universidad Autonoma de Buenos Aires, Buenos Aires, ARG; 4 Internal Medicine, Dr. Somervell Memorial C.S.I. Medical College and Hospital, Karakonam, IND; 5 Internal Medicine-Pediatrics, Geisinger Medical Center, Danville, USA; 6 Internal Medicine, St Mary Mercy Hospital, Livonia, USA; 7 Medicine, Geisinger Commonwealth School of Medicine, Scranton, USA; 8 Medicine, Guthrie Robert Packer Hospital, Sayre, USA; 9 Internal Medicine, Penn State University College of Medicine, Milton S. Hershey Medical Center, Hershey, USA

**Keywords:** helicobacter pylori eradication, disadvantages of probiotics, advantages of probiotics, mechanism of action, efficacy of probiotics, advances, management, clinical trials, probiotics and microbiome, h.pylori

## Abstract

The global prevalence of Helicobacter pylori (H. pylori) is estimated to be around 4.4 billion, with the majority of individuals affected in developing countries. Chronic infection of the gram-negative bacterium results in several gastrointestinal pathologies such as chronic gastritis, peptic ulcer, and cancer. Probiotics compete directly with H. pylori and help restore the gut microbial environment; these living microorganisms are comparatively more effective than the standard triple antibiotic regimen in the management of symptoms related to the pathogenic bacteria. The need for alternative therapy is better explained by the increasing rate of antibiotic resistance and the lowering of patient compliance to the standard treatment. Adjuvant administration of probiotics to H. pylori eradication therapy is associated with a higher H. pylori eradication rate, decreased diarrhea-related treatment, less common self-reported side effects, and higher treatment compliance. Therefore, with the ongoing and future resistance to antibiotics, this systematic review aims to investigate the use and efficacy of probiotics when used alone or in conjunction with the current guideline treatment.

A literature search was conducted using Pubmed, MEDLINE, and Cochrane for peer-reviewed articles published between January 1, 2016 and April 2022. MeSH terms used were: “H. pylori,” “H. pylori and probiotics,” “Probiotics,” “H. pylori treatment,” “Mechanism of Action” with subheadings as “clinical manifestations,” “treatment,” and “diagnosis.” All literature reviews, original papers, and case reports were included. This search strategy aimed to find literature that could describe the transmission and mechanism of action of H. pylori, the current treatment guidelines, and the efficacy of probiotics in eradicating H. pylori.

## Introduction and background

The global prevalence of Helicobacter pylori (H. pylori) is estimated to be around 4.4 billion [1], with the majority affected in developing countries, where H. pylori could infect up to 80% of the population, than in developed ones [2]. In China, the prevalence of H. pylori infection in the year 2015 was 66% among rural populations and 47% in urban settings [3]. Variation in calculating the population of affected individuals depends on several demographic factors such as age, race, and socioeconomic status [2]. 

Chronic infection of the pathogenic organism results in inflammation of gastric mucosa and associated lymphatics, leading to erosion or cancer development [3]. It is also related to the development of idiopathic thrombocytopenic purpura, vitamin B12 deficiency, and iron deficiency [2]. Until recently, a triple antibiotic regimen containing omeprazole, macrolide, and penicillin-like antibiotics are used as standard therapy. Resistance of H. pylori to such antibiotics by genetic mutations has been increasingly reported in recent studies. Due to the frequent failure of current management, concomitant alternative therapies have been considered.

Probiotics are living microorganisms that can competitively inhibit H. pylori, acting as bacteriostatic while improving the gut microbiome. Lactobacilli and other such probiotics including Bifidobacterium, Bacillus licheniformis, and saccharomyces are currently in use and are proven to be effective in managing the gastrointestinal symptoms related to H. pylori. Reduction in rate of antibiotic-associated adverse events and proven rise in H. pylori eradication are mentioned in several clinical trials, despite the limitations found in literature. Therefore, with the ongoing and future resistance to antibiotics, this revision aims to investigate the use and efficacy of probiotics in treating H. pylori eradication.

## Review

H. pylori colonization

H. pylori is a motile, microaerophilic organism with several biochemical properties. The spiral bacterium has a thin peptidoglycan layer, turning it pink on Gram stain. The urease positive action helps in the survival of H. pylori in an acidic environment and promotes protein synthesis. Maintaining the gastric pH in the acidic range is necessary for mucin gel production, while alkalinity loosens the gel and enhances bacterial motility. Colonization of H. pylori in the stomach depends on the flagellar motility [4]. Initially, the bacterium reaches the gastric area and uses its flagella to swim in the less acidic environment, and adheres to the mucus layer of the stomach. The motility is impaired by specific flagellar proteins such as fliD, FlaA, and FlaB. [4,5]. Various coupling proteins, adhesion molecules, and surface receptors of gastric cells and T1pA, B, C, and D, CheA kinase are crucial for the interaction between bacteria and host [6].

Probiotic's mechanism of action

**Figure 1:**
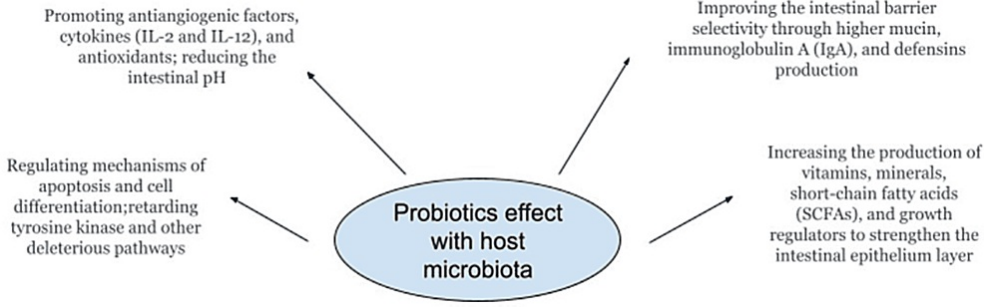
Probiotics mechanism of action on bacterial interaction with host gut microbiota

World Health Organization (WHO)/Food and Agriculture Organization (FAO) currently defines probiotics as “Live microorganisms which when administered in adequate amounts confer a health benefit on the host” [7]. Clinically, lactic acid-producing bacteria such as Lactobacillus spp, genus Bifidobacterium, Bacillus, Streptococcus [8], and Escherichia coli are effectively used as probiotics. When introduced into the human body, these organisms produce antimicrobial substances such as lactic acid, hydrogen peroxide, and bacteriocins. Lactic acid can suppress H. pylori urease activity [9]. additionally, the bacterial cell wall and its membranes are damaged by the reactive oxygen species produced by probiotics. [10]. The pharmacodynamic properties of probiotics are further explained below (Figure 1).

Adhesion of H. pylori to the gut epithelium is promoted by several bacterial surface components. Studies have mentioned that probiotics increase IgA production, strengthening the mucosal barrier against pathogens [2,3,11]. Their role against the pathogenicity of H. pylori includes competitively interacting at the microbial adhesion sites and enhancing immune response [12]. Glycolipid-binding specificity with H. pylori and probiotics are currently studied for their future application as anti-adhesion drugs in managing H. pylori-induced gastric ulcers [13]. Yang et al. found that H. pylori infection induces NFκB, IL-8, and TNF-α production in-vitro.

Clinical trials involve probiotics utilization to eradicate H. pylori

Recently, there has been more research regarding clinical trials involving probiotics (alone or in conjunction with the standard therapy) to prove the eradication of H. pylori. Ayhan et al. conducted a randomized placebo control study, where they found that significantly higher eradication rates were noted in the ERA (sequential eradication therapy) + probiotic group (86.8% vs. 70.8%, p=0.025) than in the ERA group. They concluded that adjuvant administration of probiotics is associated with numerous beneficial outcomes [14]. Shi et al. found that probiotics combined with the quadruple bismuth regimen were the best combination. Also, they listed specific probiotics such as Lactobacillus to be highly effective in achieving outcomes, making probiotics treatment strain-specific [15].

Another type of clinical study has been conducted regarding the effects of probiotics on the gut microbiota; Oh et al. observed that the usage of concomitant probiotics reduced Fermicutes while it had minimal effect on Proteobacteria. Additionally, the antibiotic resistance was controlled in the probiotic group. This effect may improve the H pylori eradication success rate [16]. Dore et al. [17] proved the potential role of Lactobacillus reuteri in H. pylori eradication by accessing the urease activity before and after 4-6 weeks of therapy which reported a significant drop in H. pylori levels after probiotic administration. Yuan et al. found that after probiotics supplemented eradication treatment, pathogenic bacteria like Fusobacterium and Campylobacter decreased. The microbial diversity was closer to H. pylori-negative subjects than in the other quadruple therapy group [18].

A prospective, multicenter placebo-controlled study performed in eight tertiary hospitals in Greece was done by Viazis et al. regarding a four-probiotics regimen combined with a standard H. pylori-eradication treatment reduces side effects and increases eradication rates. Data analysis was done for 329 patients in probiotic arm A and 335 patients in standard arm B. Now 56 (17%) patients in the probiotic cohort and 170 (50.7%) patients in the standard cohort reported with an incidence of H. pylori and severity of gastrointestinal problems. (p = 0.00001). Complete eradication of H. pylori is reported in 303 patients in probiotic arm (92.0%) and 291 patients in standard arm (86.8%), (p = 0.028).Results have shown that twice-daily probiotic supplementation reduced new-onset symptoms and severity associated with standard therapy. There was also an increase in eradication rates [19].

Another randomized clinical trial conducted by Márquez et al. on the usefulness of probiotic L. reuteri in bismuth-containing quadruple eradication therapy for infection with H. pylori was done. In this study, the effectivity of probiotics in H. pylori eradication was found to be 85%. Symptoms related to H. pylori such as abdominal pain and distension reduced by 15% in the probiotic group compared to the standard group (p < 0.001). As a result, more studies were needed to determine probiotics as adjuvant therapy in eradicating H. pylori. L. reuteri was able to reduce pain and abdominal distension [20]. Several advantages related to the concomitant usage of probiotics in H. pylori management are explained in Table 1.

**Table 1:** Advantages and disadvantages of the use of probiotics

Advantages	Disadvantages
The benefit of probiotic therapy in H. Pylori infection has shown an increased eradication rate and reduced related side effects [2].	Only a few strains can have a significant effect, so the challenge is to choose the right probiotics and the appropriate amount of them [21].
The use of probiotics may help with H. Pylori-related diseases; suppressing H. Pylori permanently with probiotics can increase the strength of the gastric mucosal barrier and compete with the bacteria for adhesion [2].	Lack of evidence, most studies made in the Asian population, and more clinical trials need to be done in patients from North America or Black individuals.
Studies have described a shallow adverse effects rate with probiotic treatment	In rare cases, probiotics have been linked to severe adverse effects such as fungemia and bacterial sepsis; potential adverse effects of probiotics must be reviewed [22].

Limitations

The only limitation of our study is the lack of risk of bias assessment for the meta-analysis studies and clinical trials included. However, the p-value of those articles included remains within the 0.5 range, making them diagnostically significant. Few clinical trials were reported with limited study samples, making it a topic of concern for future researchers.

## Conclusions

Studies have reported several probiotic uses in the management of H. pylori, either when used alone or in conjunction with the standard treatment therapy. Clinical trials have demonstrated its association with a higher H. pylori eradication rate, less common self-reported side effects, and higher treatment compliance. Nevertheless, probiotics, when used alone demonstrated to help restore the gastric dysbiosis caused by eradication therapy partially, but it has not shown any significant improvement in H. pylori eradication. Not to mention that only a few strains can significantly affect the eradication therapy, making it strain and dose-specific. Numerous meta-analyses have been conducted in the recent past to prove the effectiveness of probiotics in treating H. pylori disease. However, most of the studies were conducted in the Asian population, and more clinical trials need to be done in patients from North America or Black individuals. We also insist on future research surveys on probiotics, considering the numerous advantages and limited literature available in this field. Moreover, further applications of probiotics in recombinant technology for treating antibiotic resistance could also be explored.
